# From Garden to Pillow: Understanding the Relationship between Plant-Based Nutrition and Quality of Sleep

**DOI:** 10.3390/nu16162683

**Published:** 2024-08-13

**Authors:** Neslihan Arslan, Eda Bozkır, Tevfik Koçak, Meleksen Akin, Birsen Yilmaz

**Affiliations:** 1 Department of Nutrition and Dietetics, Faculty of Health Sciences, Erzurum Technical University, Erzurum 25050, Türkiye; akdeniz.neslihan91@gmail.com; 2 Burhaniye Chamber of Commerce, Safe Food Analysis and Export Support Center, Balıkesir 10700, Türkiye; eda.bozkir@gmail.com; 3 Department of Nutrition and Dietetics, Faculty of Health Sciences, Gümüşhane University, Gümüşhane 29100, Türkiye; dyt_tevfik@hotmail.com; 4 Department of Horticulture, Iğdır University, Iğdır 76000, Türkiye; akinmeleksen@gmail.com; 5 Department of Nutrition and Dietetics, Faculty of Health Sciences, Çukurova University, Adana 01330, Türkiye

**Keywords:** sleep, plant-based, vegan, diet, sleep quality, melatonin, tryptophan

## Abstract

The effect of diet on sleep quality has been addressed in many studies; however, whether/how plant-based diets (PBDs) impact sleep-related parameters has not been explored in detail. This review aims to give an overview of the components of PBDs and the possible mechanisms through which PBDs may improve sleep quality. Studies have indicated that diets such as PBDs, which are typically high in fruits, vegetables, nuts, seeds, whole grains, and fiber, are associated with better sleep outcomes, including less fragmented sleep and improved sleep duration. Several mechanisms may explain how PBDs impact and/or improve sleep outcomes. Firstly, PBDs are characteristically rich in certain nutrients, such as magnesium and vitamin B6, which have been associated with improved sleep patterns. Secondly, PBDs are often lower in saturated fats and higher in fiber, which may contribute to better overall health, including sleep quality. Additionally, plant bioactive compounds like phytochemicals and antioxidants in fruits, vegetables, and herbs may have sleep-promoting effects. According to available data, PBD and Mediterranean diet elements promise to enhance sleep quality; however, it is crucial to note that diets should be customized based on each person’s needs.

## 1. Introduction

Across the course of human history, various societies have included diets centered around plants. During ancient Greek times, the philosopher Pythagoras praised the advantageous effects on health of a diet centered around plants. This type of nutrition was widely referred to as the Pythagorean Diet until the 1800s. Currently, plant-based diets (PBDs) continue to be widely favored. The definition of a PBD can vary significantly, ranging from the complete exclusion of all animal products to a diet that mostly consists of vegetables, fruits, fruit juice, grains, and beans, and also fish, poultry, and yogurt [1,2]. The phrases “plant-based diet” and “vegan diet” are used interchangeably, meaning that a plant-based diet avoids animal meats and animal-derived products. However, other sources state that a plant-based diet does not equate to “being vegetarian” or vegan. This discrepancy is why the definition of a plant-based diet leads to controversy [3]. Nevertheless, diets with moderate amounts of animal sources have been cited as examples of healthy plant-based diets [4]. The Mediterranean diet stands apart from other healthy diet patterns due to its distinctive inclusion of nuts, olive oil, and the moderate use of red wine, particularly during meals. However, it may still be classified as a primarily plant-based diet [5,6].

The prevalence of vegan diets among Americans experienced a remarkable 600% surge between 2014 and 2018 [7,8]. American dietary guidelines prioritize nutritional patterns based on mounting evidence linking them to a decreased risk of chronic disease [9]. Sustainable diets maximize the efficient use of human and natural resources. These diets also prioritize the preservation and protection of biodiversity and ecosystems. Additionally, sustainable diets should be culturally accepted, easily available, economically viable, and inexpensive in addition to providing appropriate and balanced nutrition, ensuring food security, and promoting healthy living [10]. Hence, it is crucial to adopt sustainable dietary patterns to retain the optimal health of individuals [11,12]. In addition to diet and nutrition, sleep is another crucial aspect that promotes health. Sleep is an active physiological activity vital for living and typically occupies one-third of our lives. It plays a significant role in maintaining physical, mental, and emotional health [13]. 

Sleep is crucial for the overall health of individuals across different age groups, including children, adolescents, and adults. Sufficient sleep duration plays a significant role in cognitive function, mood regulation, and mental health, as well as cardiovascular, cerebrovascular, and metabolic health [14]. Optimal sleep necessitates sufficient duration, suitable timing, consistent patterns, the absence of sleep problems, and high quality [15]. The American Academy of Sleep Medicine (AASM) and the Society for Sleep Research (SRS) have jointly recommended that individuals should consistently sleep for at least seven hours every night to maintain optimal health, but individual requirements may differ [16]. Multiple studies indicate that the caliber of an individual’s sleep, as opposed to its length, is connected to their health condition [17,18]. Sleep quality refers to an individual’s level of contentment with their sleep experience. It encompasses various factors like the ease of falling asleep, the ability to stay asleep, the duration of sleep, and the feeling of being refreshed upon waking up [19]. Deficiencies in sleep quality, inadequate sleep duration, and disturbances in the body’s natural sleep–wake cycle are associated with an increased risk of cardiometabolic diseases and higher levels of abdominal fat biomarkers. These factors contribute to the development of non-communicable diseases like cardiovascular diseases, diabetes, obesity, and cancer, and result in a decline in overall health [20]. The incidence of sleep disorders or diminished sleep quality is steadily rising. The National Health Interview Survey found that 14.5 percent of individuals reported problems getting to sleep and 17.5 percent had issues remaining asleep. Having problems falling asleep became less common among adults as they got older, but remaining asleep became more of a problem. Men were less likely to have problems getting to sleep and staying asleep than women were [21]. In 2020, an Australian study with 836 participants found that 41% of women and 42% of men suffer from sleep issues. Moreover, half of the 5021 people surveyed in a separate Turkish study reported experiencing some kind of sleep disruption [22]. A separate study revealed that a significant proportion of elderly individuals reported experiencing sleep difficulties or obtaining less sleep than what is considered ideal for optimal sleep [23].

Similar to sleep, the act of consuming food is the most essential physiological and metabolic requirement for sustaining life. Enhancing the variety of meal patterns, improving food quality, and practicing chrono-nutrition can enhance sleep quality [24]. Observational studies conducted in different countries have consistently found a positive correlation between sleep quality and adherence to plant-based diets (PBDs). These studies have also shown that individuals who consumed higher amounts of fruits and vegetables had a lower dietary inflammatory index, while those who consumed diets with higher energy density, added sugar, and caffeine tended to have poorer sleep quality. Additionally, decreased intake of dairy products and unsaturated fats has also been associated with better sleep quality [25,26]. 

Plant-based foods that enhance the production of serotonin and melatonin, as well as tryptophan, which plays a crucial role in sleep metabolism, are highly beneficial for improving sleep quality [24]. A study was conducted on 7987 persons aged 20–74 years in the suburbs of Shanghai. The study found that consuming energy-dense foods and having a poor diet were linked to lower sleep quality. Findings: Among people living in the suburbs of Shanghai, adopting healthier dietary patterns and reducing beverage consumption were linked to improved sleep quality [27]. According to the Energy Balance Study (EBS), a more pro-inflammatory diet is indicated by higher dietary inflammation index scores, which, in turn, are associated with increased alertness after sleep start, poorer sleep efficiency, a later bedtime, and later awakening times [28]. Another study found that eating a high-protein diet with the necessary amino acids, low-glycemic index (GI) meals, and specific fruits rich in antioxidants may help with sleep quality, as might meal scheduling with tactics like chrono-nutrition [29]. According to a narrative review that looked at twenty human studies (6 observational and 14 interventional), a diet high in simple carbs and saturated fat was linked to worse sleep quality, whereas a diet high in complex carbs (like fiber), healthy fats (like unsaturated fats), and protein was linked to better sleep quality [30]. Another systematic review of dietary pattern research analyzed 23 studies that investigated the correlation between adherence to the Mediterranean diet and several sleep parameters, including sleep quality, sleep duration, daytime drowsiness, and symptoms of insomnia. A correlation has been established between following the Mediterranean diet and both the general quality of sleep and other sleep metrics [31].

There is increasing evidence that suggests dietary patterns and food quality play a crucial role in promoting healthy sleep quality. However, further studies are needed to validate these findings [32]. The rising popularity of plant-based nutritional patterns has a significant impact on improving both diet and sleep quality, as well as on promoting sustainable nutrition. These patterns are characterized by their high fiber content, melatonin precursors, isoflavones, and positive effects on the intestinal microbiome, making them crucial for ensuring human health. Little is known about how PDBs affect sleep health, despite the growing body of evidence supporting PDBs for lowering chronic illness and improving general health. This review aims to thoroughly describe the correlation between PBD patterns and the quality of sleep utilizing human studies. 

## 2. Methods

This article is a narrative review that aims to thoroughly investigate the correlation between PBD patterns and the quality of sleep health to provide evidence-based nutrition recommendations. 

A detailed literature search was conducted between April and June 2024. Google Scholar, PubMed, and Scopus were used for the literature search. The literature review was made using specific keywords, including “plant-based diet” OR “plant-based diet index” OR “vegetarian diets” OR “vegan” AND “sleep” and “sleep quality” OR “sleep characteristics” OR “sleepiness” OR “sleep apnea” OR “daytime sleepiness”.

Only human studies were included unless there was relevant information regarding the scope of the review. In addition to preclinical studies, studies using plant or synthetic nutritional supplements to examine the effect on sleep quality were excluded.

For the selected studies, the authors, year of publication, methodology, discussions, and conclusion parts were scanned in detail and the articles were classified according to thematic categories, including “nutritional components existing in plant-based diets”, “key nutrients related with the sleeping quality”, “common plants which are used to improve sleep quality”, “biochemical pathways and/or constituents in humans which regulates sleep cycle and quality”, and “human studies on sleep quality and plant-based diets”. Later, the information derived from the articles was gathered and expressed in the most inclusive way under each title of the review. Human studies are summarized demonstrating the study design, participants’ information, sleep quality measurement, diet-related variables, and the main outcomes of the study in Table 1. Important findings and significant relationships between the parameters are discussed in the other sections of the review. 

## 3. The Relationship between Plant-Based Diets and Sleep Quality and Overall Health 

Vegetarians are individuals who consume plant-based foods, contrary to non-vegetarians [33]. Vegetables, fruits, nuts and seeds, mushrooms, legumes, whole grains, oils, and beans are the base of a PBD, and the consumption of these foods provides high levels of antioxidants, vitamins (higher amounts of vitamins C and E and folate), minerals, dietary fiber, resveratrol, polyphenols, melatonin precursors, isoflavones, anti-inflammatory factors, and omega-3 fatty acids [34,35]. The nutrients in the PBD fight against inflammation in the body and decrease the risk of cardiovascular diseases (CVDs) and insulin resistance, lowering blood lipids (especially LDL-C) [36]. PBDs also seem to improve sleep quality, reduce the risk of mental health disorders, and decrease cognitive decline by ageing [37]. A cohort study with 135,335 participants from 18 different countries indicated that consuming more fruits, vegetables, and legumes was associated with a reduced risk of non-CVD and total mortality levels. This effect was more evident with the consumption of three or four servings of fruits, vegetables, and legumes (375–500 g/day) each day [38]. Interestingly, the sleep duration of the individuals after moving to a PBD was found to increase from 6.3 to 6.5 during the weeknights and from 6.9 to 7.3 during the weekend nights [39].

There are many advantages to following a PBD. For instance, plant-based foods contain fewer calories and fat compared to animal-based foods [35]. In a study conducted to determine the difference between a high PBD and a low PBD, the high PBD was found to contain more energy, fat, protein, cholesterol, fiber, sodium, calcium, vitamins C and D, and flavonoids compared to the low-PBD group. The high-PBD group had a lower risk of metabolic syndrome, waist circumference, hyperglycemia, hypo-HDL-cholesterolemia, and hypertriglyceridemia [40]. Another important aspect of a PBD is its low-fat content [35]. The consumption of dietary fiber is known to have a positive effect on the gut microbiome. Dietary fiber affects the gut microbiota by modifying the Prevotella/Bacteroides ratio and increasing short-chain fatty acid-producing bacteria [41]. Recent studies indicate that a plant-based diet could potentially enhance the variety of beneficial bacteria in the gut. Nonetheless, further investigation is required to elucidate the intricate relationships among nutrition, the microbiome, and health outcomes due to their complexity and individual differences. [42,43]. To benefit from the positive health effects, it is recommended to continue a PBD for at least three or six months [37].

Grain legumes are a valuable source of proteins, essential amino acids, dietary fiber, minerals, and vitamins, making them a critical component of a diverse and healthy PBD [44,45]. The trend of shifting from a meat-based to a plant-based eating style has increased the demand for legume proteins, posing a challenge for researchers and the food industry. They also provide essential minerals like iron, magnesium, and potassium, which are necessary for overall health. Legumes are rich in vitamins, including folate, thiamine, and vitamin B6, essential for various bodily functions [46].

In a PBD, beans and legumes are the base staples that support health and prevent diseases due to their insoluble and soluble fibers, vitamins, minerals, and other bioactive compounds. Consumption of beans in the diet is known to have protective effects on cardiovascular, metabolic, and colon-related chronic diseases. Moreover, due to the fibers they contain, regular consumption of beans can help prevent diseases and factors such as excess body weight, gut microbiome health, and mild inflammation [47].

Nuts, especially walnuts (*Juglans regia* L.), almonds, and hazelnuts, contain high levels of fatty acids, including mono- and polyunsaturated fatty acids, and they are beneficial for human health with moderate consumption. Nuts also contain essential minerals such as calcium, magnesium, manganese, phenolic acids, and flavonoids. Consumption of nuts in the vegetarian diet gives satiety and energy, emphasizing that their bioavailability can be increased during vegan milk production [48]. 

Vegetable protein cannot be considered as high-quality as animal protein due to its variety and amount of amino acids. However, the bioavailability can be increased by mixing cereals with legumes [49]. Vegetable protein consumption also seemed to lower the risk of CVD and mortality rates. For this reason, exchanging meat protein with plant protein is suggested to increase longevity [50,51]. Furthermore, the transition from animal- to plant-based protein foods is desirable for various reasons, including environmental stability, ethical considerations, food affordability, and greater food safety [45]. A replacement of 5% of the energy taken from animal protein with plant-based protein results in a 23% lower risk of type-2 diabetes mellitus [52]. 

Plant-based diets can lack certain nutrients if not designed carefully. Zinc, calcium, iron, vitamin D, omega 3 fatty acid (DHA), and vitamin B12, especially, are the most important nutrients that can cause nutritional deficiencies in humans [53,54]. Calcium is critical for bone health and can be obtained from many plant sources, especially leafy greens. Iron is necessary for oxygen transport and can be found in plant foods like legumes, whole grains, and green vegetables. However, in a PBD, consuming Fe-rich foods with vitamin C is crucial to increase iron absorption. Vitamin D is important for bone health and can be obtained through sun exposure or fortified plant-based sources like plant-based milk and cereals. Omega-3 fatty acids, especially DHA, are crucial for healthy brain functions, and these can be obtained from plant sources such as flaxseeds and nuts. Vitamin B12 is a principal component in animal foods, so supplementation is suggested for individuals following a PBD [53]. Legumes may contain some antinutritional factors, such as phytates and lectins, which may increase gut permeability, causing a “leaky gut” [55]. In a study with 80 participants (51 vegans and 29 non-vegans), vegans were found to have deficiencies in certain nutrients, such as omega-3 long-chain polyunsaturated fatty acids, vitamin D, calcium, sodium, and iodine. However, vegans had healthier lipid profiles and blood pressure, indicating no observed difference in HDL-C [56].

## 4. Key Nutrients Associated with Sleep Quality

### 4.1. Tryptophan

Tryptophan is produced mainly by bacteria, fungi, and plants. They can synthesize it from compounds such as phosphoenolpyruvate. However, animals lack this enzyme, and they have to take tryptophan from outer sources [57]. Tryptophan is an amino acid that is the precursor of melatonin and serotonin. It indirectly affects sleep quality [58,59]. It is also a precursor for nicotinamide (vitamin B_6_), tryptamine, kynurenine, 3-hydroxykynurenine, and quinolinic and xanthurenic acids [59].

Major dietary sources of tryptophan include dairy products, meat, fish, eggs, bananas, oats, pumpkin and sesame seeds, chocolate, dried dates, soy, tofu, tree nuts, and peanuts. The estimated dietary requirement of tryptophan for adults is approximately 280–355 mg per day [60]. From all these sources, it is taken in the form of L-tryptophan [57].

The studies show limited results for the effect of L-tryptophan on sleep problems. The effects of daily doses (<1 g and ≥1 g) on sleeping patterns were assessed. According to the meta-analysis, a dose higher than ≥1 g of tryptophan supplementation had only a shorter wake-after-sleep onset. However, no other positive effects were observed in other sleep components [61]. In other research, the relationship between tryptophan intake and sleep quality was investigated among 122 university students aged between 22 and 25. The results showed no significant relationship between tryptophan consumption and sleep quality [62]. Accordingly, for 22 women who had symptoms of fibromyalgia, a Mediterranean diet enriched with magnesium, together with the tryptophan supplementation, did not affect sleep quality. The experimental group was given a Mediterranean diet enriched with tryptophan and magnesium (60 mg of tryptophan and 60 mg of magnesium) and the control group was given only the standard Mediterranean diet for 16 weeks. It was concluded that a tryptophan- and magnesium-enriched Mediterranean diet helped reduce anxiety symptoms, mood disturbance, eating disorders, and dissatisfaction with body image but did not improve sleep quality in women with fibromyalgia [63]. 

Melatonin (n-acetyl-5-methoxytryptamine) is a neurohormone and a small lipophilic molecule mainly secreted by the pineal gland during the nighttime [64]. It informs the body about the daily cycle of light and darkness [59]. Its precursors are tryptophan and serotonin. Melatonin production is known to decrease with the ageing process [24,65].

Plant-rich foods can enhance melatonin production, and they contain high amounts of melatonin (especially tomatoes, olives, barley, rice, and walnuts) compared to animal-based foods [59,66]. However, its amount can change from picograms to micrograms per gram of plant tissue. Also, its concentration may differ according to species and within varieties of the same species. For instance, the Nebbiolo and Croatina species of grapes have 0.8–0.9 ng/g melatonin. However, Cabernet Franc contains 0.005 ng/g of melatonin. Coffee beans also contain high melatonin concentrations (about 40 µg/cup) [65,67]. However, due to caffeine’s inhibitory effect on the levels of melatonin, consuming coffee is not beneficial for the sleep cycle [24,59]. Fatty acids can affect the melatonin levels. It was found that diets poor in omega-3 fatty acids negatively influence melatonin secretion at night, possibly because the pineal gland contains a significant level of omega-3 and omega-6 fatty acids, specifically arachidonic and docosahexaenoic acids [68].

Apart from the diet, exogenous melatonin is being used to improve sleep quality in children, adults, and the elderly. The effective doses were 0.5–3 mg in children, 3–5 mg in adolescents, 1–5 mg in adults, and 1–6 mg in elderly people. No severe side effects or dependence were observed in the clinical trials [64]. The sleep-improving effects of exogenous melatonin were confirmed in adults with respiratory diseases [69], shift workers suffering from sleep disorders [70], individuals with autistic spectrum disorder [71], and children/adolescents and adults with mental or sleep disorders [72]. Its positive effect on improving sleep was also demonstrated in individuals with chronic health conditions [73,74,75,76].

### 4.2. Magnesium

Magnesium is one of the most abundant minerals in the human body. It is a co-factor in approximately 300 metabolic reactions, including melatonin production [77,78]. It is thought to regulate sleep through its interaction with glutamatergic and Gamma-Aminobutyric Acid (GABA). Furthermore, binding to GABA receptors and activating them indirectly causes a relaxation in the activity of the central nervous system [78]. Magnesium can also decrease the serum cortisol concentration, which may contribute to improved sleep patterns [77,79].

The studies regarding the relationship between magnesium and sleep quality are still limited, and further long-term research is essential to understand the mechanism [80]. According to the project Coronary Artery Risk Development in Young Adults (CARDIA) which included 3964 participants, magnesium was found to have a positive effect on sleep parameters. The subjects within the highest quartile of magnesium intake also had better sleep quality [78]. According to evidence, 500 mg magnesium supplementation for eight weeks significantly increased the sleep duration and decreased the sleep latency in the elderly [77]. In addition, magnesium deficiency also decreased the plasma melatonin levels in rats, which may cause sleeplessness [81]. However, magnesium intake did not affect the sleep quality of individuals with depressive disorder [78].

Airline pilots are known to be vulnerable to sleep disturbances. In a study with the participation of 100 airline pilots, the relationship between the 25-hydroxyvitamin D, Ca^2+^, and Mg^2+^ levels were assessed. There were no significant differences in the serum levels of 25-hydroxyvitamin D between the two groups. Also, there was no observed vitamin D deficiency in either group. However, those with lower sleep quality also had lower levels of Mg^2+^ and Ca^2+^ compared to the group with good sleeping quality (*p* < 0.001). In this research, Mg^2+^ and Ca^2+^ are considered good indicators for assessing the sleep quality [82].

With patients who were going to have open heart surgery, magnesium supplementation showed a positive impact on sleep quality [83]. In a parallel randomized clinical trial, 64 women with Polycystic Over Syndrome (PCOS) were randomly and equally assigned to a magnesium group (250 mg magnesium oxide/day) and placebo group (n = 32) for ten weeks. In this research, magnesium supplementation demonstrated no significant effect on the sleep quality of individuals [84]. However, in a cross-sectional study concluded with 175 women (18 and 40 years old), a positive correlation between the sleep quality and serum magnesium levels (in serum) was observed [85].

### 4.3. Vitamin B6

Vitamin B6 (pyridoxine) is a water-soluble vitamin and plays a role in the synthesis of some neurotransmitters, such as dopamine, serotonin, glutamate, GABA, and histamine [86,87]. Whole-grain cereals are the major dietary sources of pyridoxin [86]. Its sleep-regulating effect is thought to be due to its capacity to synthesize certain neurotransmitters [87]. However, the research on vitamin B6 alone on sleep quality is still limited. There is evidence that sleep disturbances have a relationship with increased levels of proinflammatory cytokines [88]. Vitamin B6 may enhance sleep quality by reducing inflammation in the body [89]. A randomized, double-blind, placebo-controlled study evaluated the impact of poly-γ-glutamic acid (γ-PGA) and vitamin B6 on sleep quality in 47 adults. Results showed that γ-PGA and vitamin B6 significantly impacted sleep quality [86].

### 4.4. Isoflavones

Isoflavones are a group of phytoestrogens found in legumes like fava beans, soybeans, pistachios, nuts and fruits, peanuts, and chickpeas. These compounds mimic estrogen in humans. Estrogen is important, as it balances the duration and quality of sleep [90]. In a Chinese study, higher isoflavone intake was found to be associated with lesser excessively “long sleep durations” [91]. Also, when isoflavone was given to post-menopausal women with insomnia, a significant enhancement in sleep efficiency was observed [92].

## 5. Nutritional Pathways Influencing Sleep Regulation

Plant-based diets have garnered attention for their potential impact on sleep regulation, particularly through the ingestion of tryptophan-rich foods, which serve as precursors for serotonin and melatonin synthesis [61,93]. Serotonin, a neurotransmitter crucial for sleep–wake cycle modulation, is synthesized from tryptophan in the brain. Moreover, melatonin, primarily synthesized by the pineal gland, plays a pivotal role in circadian rhythm regulation [59]. Edible plants, including whole grains, nuts, and cherries, are significant sources of tryptophan, while the Mediterranean diet, characterized by plant-based consumption, may enhance the intake of sleep-promoting compounds such as tryptophan and melatonin [93]. Additionally, isoflavones found in soybeans and legumes, prevalent in vegetarian diets, have been linked to improved sleep quality, possibly through their influence on serotonin levels and insulin-like growth factor-1 (IGF-1) production [94]. Inflammatory cytokines, nitric oxide production, and GI modulation associated with PBDs further enhance sleep quality [95]. Human studies have shown positive associations between healthful PBDs and sleep quality across various age groups, while unhealthy PBDs may inversely affect sleep quality [95]. However, inconclusive results regarding the impact of PBDs on sleep quality have also been reported [93]. Overall, these findings underscore the potential benefits of PBDs in promoting better sleep quality, though further research is warranted to elucidate the mechanisms and optimize dietary interventions for sleep improvement.

### 5.1. Tryptophan as a Mediator in Sleep Regulation 

Plant-based proteins are rich in tryptophan, a precursor of melatonin and serotonin (5-hydroxytryptamine, 5-HT), which primarily regulate sleep [93]. When tryptophan is ingested in the diet, it is transported to the brain and converted into the sleep-regulating neurotransmitter serotonin. Under normal conditions, tryptophan hydroxylase, the rate-limiting enzyme that converts tryptophan to serotonin, is not completely saturated. When tryptophan saturates this enzyme, tryptophan levels in the brain increase, and serotonin synthesis increases. This process induces sedation [61]. Serotonin is a wide-projecting, modulatory neurotransmitter generated by the brainstem’s raphe nuclei and midbrain [96], and it is an important neurotransmitter responsible for the sleep–wake cycle in the body [97]. Although it is known that serotonin regulates both sleep and wakefulness, its role is not fully understood [61,96]. Additionally, through the Mediterranean diet, known to be plant-based, the consumption of sleep-promoting compounds, including tryptophan and melatonin, could increase.

Vitamin B6 is also very important in the mediating effect of tryptophan on sleep. Vitamin B6, known as pyridoxine, is essential for the conversion of tryptophan into serotonin. 5-Hydroxytryptophan (5-HTP), an intermediary in this procedure, is transformed into serotonin by an enzyme known as aromatic L-amino acid decarboxylase (AADC) [24,98].

Studies have found a positive relationship between low calcium intake and poor sleep quality [99,100]. The association between poor sleep quality and low calcium intake may be due to the role of calcium in the central nervous system. Lower levels of calcium intake have been associated with several mood disorders, such as depression and anxiety, which are associated with poor sleep [101,102]. The importance of calcium in sleep quality lies in its role in helping the brain use tryptophan to synthesize melatonin [103].

**Figure 1:**
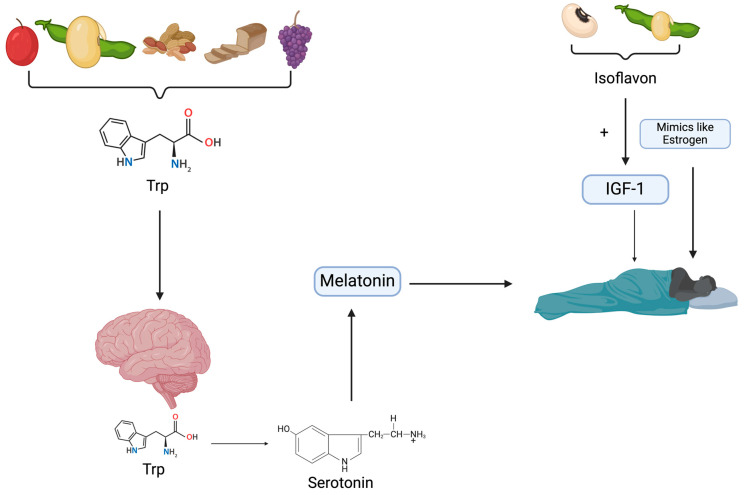
Illustration of how edible plants promote sleep regulation (Trp: tryptophan; IGF-1: insulin-like growth factor-1).

Edible plants such as whole grains, nuts, and cherries are good sources of tryptophan [104]. Additionally, through the Mediterranean diet, known to be plant-based, the consumption of sleep-promoting compounds, including tryptophan and melatonin, could increase [93]. Consumption of tryptophan-rich foods induces melatonin synthesis, responsible for the sleep cycle. Figure 1 shows the effects of tryptophan-rich foods on sleep regulation. 

### 5.2. Hormonal Factors

Melatonin (N-acetyl-5-methoxytryptamine) is the main hormone secreted by the pineal gland. Its synthesis is initiated by the conversion of tryptophan to 5-hydroxytryptophan, catalyzed by the enzyme tryptophan-5-hydroxylase [59]. This two-step process involves the sequential actions of two enzymes: Hydroxyindole-O-Methyl Transferase (HIOMT) and serotonin-N-Acetyl Transferase (NAT), with the latter being the limiting enzyme for the synthesis of melatonin. In the pineal gland, the mRNAs that code for these enzymes are expressed in a day–night cycle [105]. Environmental light and the body’s internal circadian clock regulate melatonin production. The primary environmental component governing its synthesis is light. Pineal melatonin levels start to rise in the late evening and peak between 2:00 and 4:00 a.m., after which they gradually decrease to lower levels during the day. Melatonin levels during the day are hardly noticeable. Apart from natural daylight, artificial indoor lighting can also be intense enough to inhibit melatonin secretion during the night [59].

The melatonin content in edible plants has garnered attention in nutritional sciences in recent years. It has been shown that fruit and vegetable intake is related to better sleep quality [106]. Consumption of Jerte Valley cherry cultivars has been shown to impact the sleep–wake cycles of middle-aged and older participants positively. While this study also highlighted cherries’ high antioxidant content, it suggested that the presence of tryptophan, serotonin, and melatonin in them may have contributed to improved sleep quality [107]. In another study, grape seed proanthocyanidin extract treatment maintained nocturnal melatonin levels [108].

Magnesium as a micronutrient plays a pivotal role in melatonin synthesis. Through its interaction with glutamatergic and GABA, it helps to control sleep [77,80]. 

Vegetarian and vegan individuals who follow a PBD, especially, are known to consume soybeans and legumes instead of animal-derived protein [94,109]. Soybean and legumes are rich in isoflavones, which are known to be related to sleep quality [91,92,110]. Isoflavones in the human body act like estrogen, which is known to be related to better sleep quality by affecting serotonin levels [90]. Serotonin is responsible for the sleep–wake cycle, as mentioned before [97]. Additionally, isoflavone intake is related to a greater increase in IGF-1 [111]. IGF-1 was related to improvements in sleep–wake disorders [112].

### 5.3. Gut Microbiota 

Research has reported a relationship between gut microbiota and sleep quality [113]. Individuals with abnormal sleep patterns have been shown to have gastrointestinal dysfunction [114]. It can be said that plant-based diets known to be rich in fiber have positive effects on microbiota [115]. A previous study stated that improvements in sleep quality after having a PBD are thought to be related to changes in the gut microbiome, such as a lower ratio of Firmicutes to Bacteroidetes, which could, in turn, improve sleep quality [95,116]. Total microbiome diversity was positively associated with sleep efficiency and the total sleep time, while it was negatively associated with wake-after-sleep onset in human subjects [117].

### 5.4. Anti-Inflammatory Mechanisms

Inflammatory cytokines such as IL-1 β, TNF-α, CRP, and IL-6 have been shown to play a role in sleep regulation [118,119,120,121]. It was shown that poor sleep quality is associated with the saliva IL-1 β levels in young people [122]. In another study, sleep inconsistency was found to be related to inflammation [121]. 

There are a number of mechanistic hypotheses that may explain the relationship between sleep and inflammation. It is known that edible green plants are rich in nitrates. The Mediterranean diet, one of the plant-based dietary patterns, contains more nitrates than the Western-type diet [95]. When foods containing nitrates are consumed, they are known to be converted into nitric oxide. Nitric oxide may contribute to sleep quality by preventing endothelial dysfunction [123]. Another contribution of PBD to sleep via inflammation could be having a lower GI [124]. Plant-based diets are high in soluble fiber and low in GI carbohydrates (fruits, vegetables, and whole grains, such as oats and barley); these diets are characterized by low postprandial insulin secretion and a slow absorption rate from the gut, thus preventing insulin resistance and hyperinsulinemia, which are known to be responsible for inflammation [125,126].

Another nutrient in which inflammatory pathways have an impact on sleep is vitamin D. Insufficient levels of 25(OH)D adversely impact sleep by triggering the activation of proinflammatory agents, including interleukin-1 (IL-1), tumor necrosis factor-alpha (TNF-α), and prostaglandin D2 (PGD2) [127]. According to reports, IL-1 and TNF-α have a direct influence on the sleep–wake cycle [118,119,120,121].

### 5.5. Other

Another pathway that is effective in regulating sleep is body temperature [128]. Administering glycine orally before bedtime has been demonstrated to enhance the quality of sleep by affecting the N-methyl-d-aspartate (NMDA) receptors in the suprachiasmatic nucleus (SCN), which is responsible for regulating the body’s internal clock. This is achieved by inducing a decrease in body temperature and vasodilation [129].

## 6. Human Studies on Sleep and Plant-Based Diets

Several studies investigated the relationship between sleep quality and dietary habits (Table 1). Pourreza et al. (2021) conducted a cross-sectional study involving 390 overweight and obese women aged between 18 and 48, finding an association between lower sleep quality and unhealthful plant-based diets [130]. Another intervention study with a vegetarian group (n = 30) and a non-vegetarian group (n = 30) revealed that the vegetarian group experienced significantly better sleep [33]. A positive association between healthful plant-based diets and sleep quality was observed in a cross-sectional study with 2424 participants aged 45 years and older [104]. Similarly, positive associations were found between healthful plant-based diets and sleep quality among 1643 adolescents aged 11 and 14 years [131]. Adolescents with early bedtimes also tended to consume more fruits and vegetables, positively impacting their sleep duration [116]. Daneshzad et al. (2020) conducted a pilot intervention study with 14 patients with obstructive sleep apnea, indicating that a 21-day, whole-food, plant-based diet reduced daytime sleepiness [132]. Conversely, it was found that individuals with a high unhealthful PBD index had the worst sleep quality, while those with a high healthful plant-based diet index had the best sleep quality, among 230 diabetic women [133]. Participants were categorized as vegan, vegetarian, pescatarian, or omnivores according to Mediterranean Diet Adherence Screener (MEDAS) results, finding no significant impact of diet on sleep quality [134]. Finally, Zuraikat et al. (2020) observed a positive predictive association between adherence to the Mediterranean diet (fruit, vegetable, and legume consumption could predict higher sleep efficiency) and sleep quality among 432 women aged 20–76 in a cross-sectional study [95]. Based on current evidence, PBDs and the components of the Mediterranean diet appear promising for improving sleep quality in both healthy and unhealthy individuals. However, it is important to note that while there are numerous reported benefits of PBDs on sleep, diets should be planned based on individual requirements.

**Table 1:** Human studies on sleep and plant-based diets.

Reference	Study Design	Participants	Sleep Quality Measurement	Diet-Related Variables	Main Outcomes
[130]	Cross-sectional study	390 overweight and obese women aged 18–48	The Pittsburgh Sleep Quality Index	FFQ was obtained, and plant-based dietary scores were calculated.	Unhealthful plant-based index was found to be associated with lower sleep quality. They failed to find an association between overall plant-based dietary scores and sleep quality.
[33]	Intervention study	Vegetarian group (n = 30) was fed a vegetarian diet and non-vegetarian group (n = 30) was fed a non-vegetarian diet for three months.	The Pittsburgh Sleep Quality Index	FFQ form containing fruit, vegetable, dairy product, fish, cereal, pulse, egg, meat, fat, sweet, beverage, and nut food groups.	The vegetarian group had significantly better sleep scale scores.
[104]	Cross-sectional study	2424 participants, 45 years and older	The Pittsburgh Sleep Quality Index	Semi-quantitative FFQ was obtained, and plant-based dietary scores were calculated.	A positive association between healthful plant-based index and overall plant-based index and sleep quality was found. A negative association between sleep quality and unhealthful plant-based index.
[131]	Cross-sectional study	1643 male and female adolescents aged between 11 and14 years	Pediatric Daytime Sleepiness Scale and self-reported sleep time	KIDMED and FFQ for Italians	Adolescents who had an early bedtime were found to eat more fruits and vegetables. Consumption of fruits and vegetables positively correlated with overall and weekday sleep duration.
[116]	Pilot intervention study	14 patients who have obstructive sleep apnea with a mean age of 59.1, BMI > 22	Epworth sleepiness scale	Participants had a whole-food, plant-based diet for 21 days.	A 21-day WFPB diet intervention decreased sleepiness during the day.
[132]	Cross-sectional study	230 diabetic women	The Pittsburgh Sleep Quality Index	FFQ was obtained, and plant-based dietary scores were calculated.	It was determined that individuals with high UPDIs had the worst sleep quality, and individuals with high HPDIs had the best sleep quality.
[133]	Pilot study	The 62 individuals who participated in the study were categorized as vegan, vegetarian, pescatarian, and omnivores according to the MEDAS result.	The Pittsburgh Sleep Quality Index	Mediterranean Diet Adherence Screener (MEDAS) questionnaire	Diet was not found to be effective for sleep quality.
[134]	Cross-sectional observational study	245 community physicians	Sleep-Related Impairment—short form	FFQ was obtained.	Each 1 SD increase in the plant-based diet score was associated with a 0.71-point decrease in the SRI.
[95]	Cross-sectional study	432 women aged 20–76	The Pittsburgh Sleep Quality Index	Alternate Mediterranean (aMed) diet score	A positive predictive association was found between adherence to the Mediterranean diet and sleep quality.

PDI: plant-based diet index; uPDI: unhealthy plant-based diet index; hPDI: healthy plant-based diet index; BMI: body mass index; KIDMED: Mediterranean diet quality index; FFQ: Food Frequency Questionnaire.

## 7. Impact of Dietary Patterns and/or Meal Timing on Nutritional Pathways Influencing Sleep Regulation

Dietary patterns and meal timing play a crucial role in influencing the nutritional pathways that regulate sleep through the regulation of the circadian rhythms, epigenetic processes, and gut–microbiota interactions [135]. The timing of meals, particularly breakfast omission and nocturnal feeding, can impact metabolic responses, energy balance, and subsequent sleep cycles, highlighting the importance of nutrient timing in overall health and sleep regulation [136]. Meal timing affects human circadian rhythms, particularly glucose regulation, with minimal impact on insulin and triglyceride rhythms, suggesting a stronger influence on glucose homeostasis [137]. According to a study, skipping breakfast, having late-night snacks, and irregular meal timing were associated with poor sleep quality among university students [138]. Meal timing, particularly delaying supper close to sleep, may disrupt circadian rhythms and metabolic health, potentially influencing sleep regulation and the cancer risk through glucose and lipid metabolism pathways [139]. The findings of a study suggest that sleep timing, independent of its duration, is associated with dietary patterns, including lower fruit and vegetable consumption and higher sweetened beverage intake, influencing sleep regulation pathways [140]. There is supporting evidence as to the impact of a shorter sleep duration due to lower fruit and vegetable consumption among adolescents [141]. The macronutrient composition of the evening meal, particularly carbohydrates and high-GI foods, is associated with a longer sleep duration in early childhood [142]. Diets rich in healthy foods help to improve sleep quality, whereas diets high in processed and sugar-rich foods have been found to be associated with poorer sleep patterns [25]. Poor quality of sleep was associated with the lower consumption of healthy plant-based foods, including fruits, vegetables, whole grains, and legumes, in another study [143]. The modern dietary patterns are linked to a shorter sleep duration and higher BMI, emphasizing the importance of healthy eating habits for overall health [144]. Moreover, it was found that the nutritional balance of protein and carbohydrates influences sleep regulation by affecting glucose, glycogen, and specific amino acids, like glycine, serine, and threonine, as shown in the Drosophila model [145]. Protein and fiber intake was associated with a better sleep, while higher carbohydrate, sugar, and saturated fatty acid intake was associated with poorer sleep quality, disturbances, and fatigue [30,146]. A diet high in saturated fat is associated with a lower quality of sleep. One potential mechanism for increased saturated fat intake to negatively affect sleep quality is said to be the consumption of low amounts of carbohydrates, which are known to facilitate the maintenance of sleep [147]. Another mechanism might be related to the time of the consumption of fats. Fat consumption close to bedtime may cause gastrointestinal symptoms that disrupt sleep and increase waking. High-fat foods also decrease stomach emptying and relax the lower esophageal sphincter, which can worsen post-prandial gastroesophageal reflux. Thus, it may indirectly impair sleep quality through reflux [148]. It has been established that the consumption of dietary fiber has a beneficial impact on the gut microbiome [149].

Increasing the number of bacteria that produce short-chain fatty acids and altering the ratio of Prevotella to Bacteroides are two of the ways that dietary fiber influences the microbiota in the gut [41]. According to a previous study, improvements in sleep quality after having a PBD are likely to be associated with changes in the gut microbiome, such as a decreased ratio of Firmicutes to Bacteroidetes, which could, in turn, enhance sleep quality [95,116]. These findings emphasize the significant relationship between dietary intake, meal timing, and sleep quality, suggesting that modifying dietary patterns and meal timing could serve as non-pharmacological interventions to enhance sleep and metabolic health.

## 8. Strengths and Limitations

In this review, we aimed to elucidate the relationship between sleep quality and plant-based diets by synthesizing the existing literature. One of the strengths of this review is that it examines a good number of relevant human studies which discuss the nutrients of plant-based diets and their relationship with sleep quality. Additionally, this review distinguishes itself by examining studies that assess both the key components of PBDs and the comprehensive impact of plant-based diets, considering potential synergistic effects on sleep quality.

Given the predominance of cross-sectional studies in the literature, incorporating more intervention studies would enhance the examination of the cause-and-effect relationships. Another limitation identified in this review is the prevalent use of subjective methods to evaluate the sleep quality in the included studies. Future research should prioritize intervention studies to elucidate causal relationships. Furthermore, in future studies, it will be useful to study cohort studies with large samples using objective sleep quality methods.

## 9. Conclusions

There is an increasing demand for vegetarian food. The components of PBDs have been associated with many health outcomes. Sleep, a physiological need for human beings, plays a significant role in human health. It is known that diet has a close relationship with sleep. The current study evidence (mostly based on cross-sectional studies) has shown that PBDs may improve sleep outcomes, such as the duration and quality of sleep, in addition to having positive effects on sleep-related diseases, such as sleep apnea. One of the key mechanisms to explain how PBDs may be involved in sleep regulation is via tryptophan. Plant-based proteins are rich in tryptophan, which is a precursor of melatonin and serotonin. Furthermore, anti-inflammatory mechanisms associated with PBDs, gut microbiota, and hormonal factors are other possible mechanisms that have been reported in the studies. Despite the promising findings of the studies, it is essential to note that individual responses to dietary changes can vary. While many studies suggest a positive association between plant-rich diets and sleep, more research (especially clinical trials) is needed to understand the mechanisms and long-term effects fully. Additionally, factors such as the overall diet quality, the timing of meals, and individual differences in metabolism and gut microbiota may also influence the relationship between plant-based diets and sleep. Hence, future studies should consider all aspects to better understand the relationship between PBDs and sleep patterns to provide more comprehensive recommendations for optimizing sleep through dietary interventions.
